# High oleic soybean oil maintains milk fat and increases apparent total-tract fat digestibility and fat deposition in lactating dairy cows

**DOI:** 10.3168/jdsc.2023-0411

**Published:** 2024-03-02

**Authors:** Samantha L. Hanno, Aaron M. Hurst, Kylie Weaver, Andrew T. Richards, Maria E. Montes, Jacquelyn P. Boerman

**Affiliations:** Department of Animal Sciences, Purdue University, West Lafayette, IN 47907-2063

## Abstract

Due to the bioactive properties of oleic acid, the objective of this study was to feed high oleic soybean oil (HOSO) to lactating cows and evaluate milk production, body composition, and apparent total-tract digestibility variables. Thirty Holstein cows (n = 16 primiparous, n = 14 multiparous at 87 ± 26 DIM at the start of the trial) were used in a crossover design with periods lasting 21 d. The treatments were a control (CON) diet with no added soybean oil and a HOSO diet with 1.5% diet DM of high oleic soybean oil. Dry matter and milk production data were collected the last 7 d of each period, and milk composition was collected the last 3 d of each period. Fecal samples were collected 6 times during the last 3 d of each period. Body weights were collected on the last 3 d of each period after the morning milking and ultrasound scans of the longissimus dorsi on the last day of each period. Compared with CON, HOSO did not affect DMI, milk production, and milk component yields. However, a parity effect was observed with multiparous cows having increased DMI (5.9 kg/d), milk production (11.2 kg/d), and component yields, with no treatment by parity interactions. Milk fat concentration tended to be greater for HOSO cows. Body weight data tended to have an interaction between treatment and parity, with multiparous HOSO cows having increased BW compared with CON and no effect on primiparous cows. Similar treatment by parity effects were observed for BCS. Compared with CON, HOSO increased fat depth by 0.44 mm and apparent total-tract fat digestibility by 12 percentage units. The results of this study indicated no detrimental effects of HOSO on milk production parameters with an increase in milk fat concentration, fat digestibility, and deposition compared with a control diet.

## Highlights

• High oleic soybean oil maintained milk yield and increased milk fat concentration compared with control.
• High oleic soybean oil increased apparent total-tract fat digestibility compared with control.
• High oleic soybean oil increased backfat depth for both parity groups compared with control.
• Body weight and body condition score increased more for multiparous than for primiparous cows.

## Graphical Abstract

**Figure:**
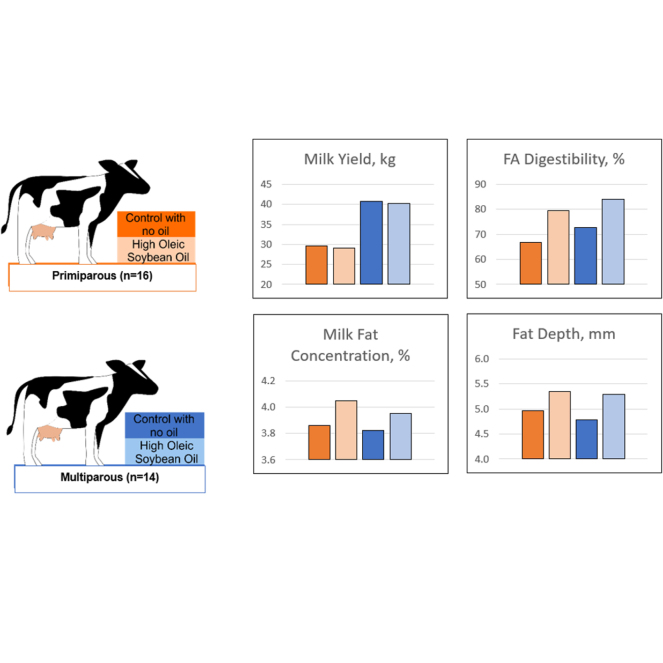
**Summary:** High oleic soybean oil was supplemented to dairy cattle to evaluate the effects on production performance and digestibility. Oleic acid supplementation had no impact on milk fat yield and increased milk fat concentration compared with a control with no added supplemental fat. Additionally, high oleic soybean oil improved apparent total tract fat digestibility and increased backfat depth of the diet compared with the control diet for both primiparous and multiparous cattle. A treatment by parity interaction was observed in other body composition variables, as multiparous cows fed supplemental high oleic soybean oil tended to weigh more and had a significant increase in body condition score, with no changes observed in primiparous cows.

Traditionally, the fatty acid (**FA**) profile of *Glycine max* (soybean) contains greater than 50% linoleic acid (C18:2; Gunstone et al., 2007; Dijkstra, 2016). Polyunsaturated FA are toxic to certain rumen microbes, leading to biohydrogenation converting PUFA to SFA (Maia et al., 2007). If excessive PUFA are fed to dairy cattle, especially in an oil form, it may cause the production of bioactive biohydrogenation intermediates that directly cause reduced milk fat synthesis known as milk fat depression (**MFD**; Bauman and Griinari, 2003). Oleic acid (*cis*-9 C18:1) has a much less potent effect on reducing milk fat synthesis (He et al., 2012; Stoffel et al., 2015) and has demonstrated positive effects on maintaining body condition and increasing milk production in dairy cattle (de Souza et al., 2019).

Progressive soybean breeding programs have led to the development of varieties with a high oleic content (Burton, 1991). These soybean varieties have increased the availability and popularity of high oleic oils to meet food processing and human health–related consumer demands (Zambelli, 2021). These high oleic soybean varieties meet the dietary needs of humans and improve the flexibility of diet formulation for dairy cattle. Weld and Armentano (2018) demonstrated that feeding high oleic soybeans, either whole or ground, increases milk fat concentration compared with conventional soybeans. Additionally, feeding trials that increase the amount of oleic acid relative to palmitic or stearic acid have shown improved fat digestibility (Prom and Lock, 2021).

The evidence of positive bioactive properties of oleic acid has increased the development of commercial fat supplements containing higher concentrations of oleic acid. However, it is unknown if feeding high oleic soybean oil (**HOSO**), without rumen protection, would result in similar production responses as those observed with feeding higher amounts of oleic acid in fat supplements. Therefore, the objective of this study was to feed HOSO to lactating cows and evaluate production, body composition, and apparent total-tract digestibility variables. Our hypothesis is that HOSO would maintain milk fat concentration, increase fat digestibility, and increase BW gain relative to a control diet.

Experimental procedures were reviewed and approved by the Institutional Animal Care and Use Committee at Purdue University, West Lafayette, Indiana (#2201002234). Thirty mid-lactation (87 ± 26 DIM; mean ± SD) Holstein cows (primiparous n = 16, multiparous n = 14) from the Purdue University Animal Sciences Research and Education Center were blocked by lactation number and milk yield, then randomly assigned to a treatment sequence in a crossover design with 21-d periods. All animals were fed a common diet during a 14-d preliminary period to obtain the baseline values used for treatment allocation.

Treatments included a control diet (**CON**) containing wheat middlings or a diet supplemented with HOSO at 1.5% of diet DM. The HOSO contained approximately 7.5% C16:0, 3.6% C18:0, 76.8% C18:1, 8.5% C18:2, 1.6% C18:3, and 2.0% other FA, and samples were analyzed at the Bindley Metabolite Profiling Facility (Purdue University, West Lafayette, IN) following the FA preparation and GC method for oil samples (Amat Sairin et al., 2022). The ingredient and nutrient composition of the diets are presented in Table 1. Diets contained similar amounts of NDF, ADF, and CP with the HOSO treatment containing 1.29 percentage units more FA, resulting in higher NE_L_ (1.70 vs. 1.65 Mcal/kg) in the HOSO versus CON diet, respectively. All animals were fed once per day between 0700 and 0800 h at 110% of expected intake with orts collected immediately before feeding. Water was available ad libitum in each tiestall. Stalls were lined with waterbed mattresses and bedded with sawdust twice per day. Cows were milked twice daily (0500 and 01600 h).

**Table 1:** Ingredient and nutrition composition of the control (CON) and high oleic soybean oil (HOSO) diets

Item	CON	HOSO
Ingredient (% of DM)		
Corn silage	26.5	26.5
Alfalfa silage	13.1	13.1
Alfalfa hay	3.5	3.5
Rye hay	2.7	2.7
Wheat straw	1.7	1.7
Cottonseed	11.1	11.1
Corn grain	21.1	21.1
Soybean meal	4.9	4.9
Expeller soybean meal^1^	4.0	4.0
Blood meal	1.2	1.2
QLF 63/43^2^	5.4	5.4
Mineral-vitamin mix^3^	3.4	3.4
Wheat middlings	1.5	
High oleic soybean oil		1.5
Nutrient analysis (% of DM unless otherwise noted)		
DM (% of diet)	55.2	55.3
NDF	28.8	28.4
ADF	19.5	20.0
CP	17.8	17.4
Ash	7.7	8.1
Fat	5.46	6.75
C16:0	1.10	1.15
C18:0	0.17	0.22
C18:1	0.95	2.17
C18:2	2.69	2.64
C18:3	0.30	0.31

1 Expeller soybean meal (SoyPlus, West Central Soy, Ralston, IA).

2 Molasses products, condensed whey, condensed soybean solubles, corn syrup, and sulfuric acid (Quality Liquid Feeds, Dodgeville, WI).

3 Mineral vitamin mix contained 28.5% calcium carbonate, 19.0% sodium bicarbonate, 10.1% salt, 7.5% urea, 6.3% calcium monophosphate, 6.3% magnesium oxide, 5.7% microbial fermentate product (Diamond V XP, Diamond V, Cedar Rapids, IA), 5.0% porcine tallow, 3.1% potassium carbonate product (DCAD Plus, Church and Dwight Co. Inc., Arm and Hammer Animal Nutrition, Ewing Township, NJ), 2.7% methionine analog product (Alimet, Novus International, St. Charles, MO), 2.5% trace mineral vitamin premix #5390, 1.5% rumen-protected methionine product (Smartamine M, Adisseo, Alpharetta, GA), 1.5% antifungal agent (ETX-5, Feedworks USA, Cincinnati, OH), and 0.3% vitamin E.

Milk yield and DMI measurements were collected daily for the duration of the experiment. Ingredient samples and TMR samples were collected once weekly and stored at −20°C until dried. On the last 3 d of each period, 50-mL milk samples were collected at each milking (d 19 to 21). For apparent total-tract digestibility estimates, fecal samples of approximately 450 g were collected 30 min before each milking on the last 3 d of each period. Feces were stored at −20°C until dried and composited by cow-period. Body weights were measured on the last 3 d of each period directly after the morning milking. Body condition scores were determined by 3 trained researchers using a 5-point scale in 0.25 increments on the last day of each period (Wildman et al., 1982) with scores averaged per animal period. Ultrasound scans above the longissimus dorsi were performed on the last day of each period using an Aloka SSD-500 ultrasound (Wallingford, CT). Three ultrasound images were captured on the right-hand side of each animal at the 12th intercostal space by a trained researcher using methods described by Schäff et al. (2013) without compression of tissue.

Samples of diet ingredients, TMR, and feces were dried in a 60°C forced-air oven for 72 h for DM determination. Composited by cow-period, dried feces samples were ground through a 1-mm screen (Retsch, GmbH, Haan, Germany). Diet ingredients, TMR, and fecal samples were analyzed by Cumberland Valley Analytical Services (Hagerstown, MD) for ash-free NDF with heat-stable α-amylase and sodium sulfate (Van Soest et al., 1991), and CP (AOAC International, 2006; method 990.03). Undigestible NDF at 240 h (**uNDF_240_**) was used as an internal marker to estimate fecal output and calculate apparent total-tract digestibility. Previous studies have determined uNDF_240_ to be a reliable estimate for indigestible NDF (Cochran et al., 1986; Palmonari et al., 2016). Milk samples were analyzed for fat, true protein, lactose concentrations, MUN, and SCC using Fourier Transform Infrared Spectroscopy (MilkScan 7 RM, Foss, Hillerød, Denmark) by Dairy One (Ithaca, NY). Yields of components and ECM were calculated by using milk yield and component concentrations for each milking and summed by day for the last 3 d of each period. Muscle and fat measurements were measured utilizing ImageJ software (National Institutes of Health, Bethesda, MD) by a single trained observer. The 3 measurements per animal period were averaged by period.

A power analysis was performed to calculate the sample size needed to detect differences in FA digestibility and milk fat concentration. With 95% confidence and 80% power, 14 animals per treatment were needed to detect a 2 percentage-unit difference in FA digestibility and 0.10 percentage unit change in milk fat concentration. To consider potential differences in response to treatment based on parity, 16 primiparous and 14 multiparous cows were enrolled in the study. All data were analyzed using the MIXED procedure of SAS (version 9.4; SAS Institute Inc., Cary, NC) with repeated measures where appropriate. The Toeplitz covariance structure was used based on minimizing Bayesian information criterion. Production, BW, and digestibility data were analyzed for normality using the Shapiro-Wilk test and were considered normally distributed. The model was Y_ijklm_ = µ + T_i_ + L_j_ + TL_ij_ + P_k_ + C_l_ + e_ijklm_, where Y_ijklm_ is the dependent variable, µ is the overall mean, T_i_ is the fixed effect treatment (i = CON or HOSO), L_j_ is the fixed effect of parity (j = primiparous or multiparous), TL_ij_ is the interaction between treatment and parity, P_k_ is the random effect of period, C_l_ is the random effect of cow (l = 1 to 30), and e_ijklm_ is the residual error. Treatment and interaction terms were considered significant at *P* ≤ 0.05, and tendencies were determined at *P* ≤ 0.10 and >0.05. All values are expressed as LSM and SEM for treatment by parity unless otherwise stated.

Due to a lack of interactions between treatment and parity for production parameters, main effects will be presented. There were no observable differences in DMI between HOSO and CON animals (*P* = 0.81; Table 2). However, multiparous animals consumed 5.9 kg/d more DMI than primiparous animals (*P* < 0.01). There was a parity effect on milk yield with multiparous cows producing 11.1 kg/d more milk than primiparous cows (*P* < 0.01) with no observed treatment effect (*P* = 0.34). These differences in milk yield and DMI by parity indicate that multiparous cows had increased feed efficiency compared with primiparous cows (*P* < 0.01). Yields of milk components were not affected by treatment (*P* > 0.05). However, milk component concentrations were affected by treatment; HOSO tended to increase milk fat (0.16 percentage units; *P* = 0.07) and protein (0.07 percentage units; *P* = 0.10) concentrations compared with CON. Additionally, MUN was greater for multiparous (*P* < 0.01) and HOSO cows (*P* < 0.01) compared with primiparous and CON cows, respectively.

**Table 2:** Dry matter intake, milk production and composition, feed efficiency (FE; ECM/DMI), BW, BCS, and ultrasound measurements for cows fed treatment diets^1^ (n = 30)

Variable	Primiparous		Multiparous		SEM	*P*-value^2^		
	CON	HOSO	CON	HOSO		TRT	Parity	TRT × parity
DMI, kg/d	24.5	24.6	30.5	30.2	0.33	0.81	<0.01	0.47
Milk yield, kg/d	29.6	29.0	40.7	40.2	0.56	0.34	<0.01	0.91
ECM,^3^ kg/d	31.5	31.6	42.2	42.8	1.44	0.58	<0.01	0.67
Milk composition								
Fat yield, kg/d	1.12	1.15	1.51	1.55	0.05	0.20	<0.01	0.73
Protein yield, kg/d	0.95	0.94	1.23	1.26	0.02	0.66	<0.01	0.47
Lactose yield, kg/d	1.48	1.44	1.98	1.97	0.04	0.47	<0.01	0.74
Fat, %	3.86	4.05	3.82	3.95	0.20	0.07	0.41	0.76
Protein, %	3.24	3.31	3.12	3.19	0.04	0.10	<0.01	0.94
Lactose, %	5.03	5.04	4.97	4.95	0.01	0.81	<0.01	0.53
MUN, mg/dL	11.7	12.4	13.1	14.9	0.33	<0.01	<0.01	0.09
FE (ECM/DMI)	1.27	1.27	1.40	1.39	0.05	0.98	<0.01	0.88
BW, kg	595	590	651	700	15.6	0.15	<0.01	0.08
BCS^4^	2.97	2.98	2.79	3.03	0.05	0.01	0.20	0.03
Muscle depth,^5^ cm	4.68	4.49	4.27	4.53	0.14	0.80	0.18	0.10
Fat depth,^5^ mm	4.97	5.34	4.79	5.29	0.20	0.03	0.54	0.75

1 Treatments contained 1.5% wheat middlings (control, CON) or 1.5% high oleic soybean oil (HOSO) fed to 16 primiparous and 14 multiparous dairy cattle in a crossover design.

2 *P*-values associated with treatment effects (CON vs. HOSO), parity effects (primi- vs. multiparous), and the interaction between treatment and parity.

3 ECM = (0.327 × kg of milk) + (12.95 × kg of milk fat) + (7.20 × kg of milk protein).

4 BCS using 5-point scale; 1 = a very thin cow with no fat reserves and 5 = a severely over-conditioned cow.

5 Muscle and fat depth measurements taken from ultrasounds above the longissimus dorsi.

A tendency for a treatment by parity interaction was observed for BW with multiparous HOSO cattle having increased BW compared with CON and no differences observed in primiparous cattle (*P* = 0.08; Table 2). A parity by treatment interaction was observed for BCS, with greater BCS observed in HOSO multiparous cows but no differences between treatments for primiparous cows (*P* = 0.03). Fat depth measured by ultrasound imaging demonstrated that HOSO increased backfat for both primiparous and multiparous cows (*P* = 0.03). Additionally, HOSO treatment increased change in BCS through the period compared with CON treatment (*P* = 0.03).

There were no differences in apparent total-tract DM digestibility for treatment (*P* = 0.80) or parity (*P* = 0.60; Table 3). The HOSO treatment tended to reduce apparent total-tract NDF digestibility (*P* = 0.08). There was an interaction between parity and treatment for apparent total-tract CP digestibility (*P* = 0.05) with HOSO reducing CP digestibility in primiparous cows with little effect in multiparous cows. The HOSO treatment increased apparent total-tract fat digestibility estimates (*P* < 0.01), and multiparous cows had increased digestibility compared with primiparous cows (*P* ≤ 0.01).

**Table 3:** Apparent total-tract digestibility estimates for cows fed treatment diets^1^ (n = 30)

Variable	Primiparous		Multiparous		SEM	*P*-value^2^		
	CON	HOSO	CON	HOSO		TRT	Parity	TRT × parity
DM, %	66.6	65.8	66.1	66.7	0.48	0.80	0.60	0.15
NDF, %	41.1	38.7	41.0	40.0	1.00	0.08	0.56	0.45
CP, %	65.9	62.5	65.9	65.5	0.76	0.01	0.05	0.05
FA, %	66.8	79.6	72.8	84.1	1.85	<0.01	<0.01	0.67

1 Treatments contained 1.5% wheat middlings (control, CON) or 1.5% high oleic soybean oil (HOSO) fed to 16 primiparous and 14 multiparous dairy cattle in a crossover design.

2 *P*-values associated with treatment effects (CON vs. HOSO), parity effects (primiparous vs. multiparous), and the interaction between treatment and parity.

Unlike conventional soybean oil supplementation (Boerman and Lock, 2014), oil from high oleic soybeans did not reduce milk fat concentration. Feeding high enough quantities of HOSO may have increased the amount of oleic acid reaching the small intestine based on the observed increase in apparent total-tract fat digestibility. However, we did not measure duodenal flow to be able to say this definitively. The improvement in apparent total-tract digestibility of FA with no change in ECM can be partially explained by differences in fat depth accumulation between HOSO and CON treatments across both parity groups. Multiparous cows responded differently to HOSO supplementation than primiparous cows in BW and BCS with no changes seen in primiparous cows. We are unaware of other research that has reported parity effects to high oleic soybean products.

Fat sources have previously been supplemented to dairy cattle to provide a more energy-dense ration. However, feeding a higher fat diet has shown to decrease FA digestibility indicating that increasing the FA concentration in the diet may overwhelm the small intestine and its ability to digest lipids (Palmquist, 1991; Boerman et al., 2015). These negative effects on digestibility may be more frequently observed when feeding fats higher in SFA, specifically stearic acid (Boerman et al., 2015, 2017; Western et al., 2020a). This is contrary to what we observed when oleic acid was supplemented, as HOSO cows had increased fat digestibility compared with CON. The increased fat digestibility observed in this study is similar to previous research in oleic acid supplementation (Western et al., 2020b; de Souza et al., 2021a; Prom and Lock, 2021). Due to the supplementation of oleic acid being in oil form, which is typically readily available for biohydrogenation, the form may have also aided in the fast passage rate of FA from the rumen, leading to higher oleic acid availability in the small intestine (Daley et al., 2020).

On the other hand, we observed no change in DM and a decrease in NDF digestibility in HOSO cows compared with CON. This is contrary to de Souza et al. (2021a), who reported that an increased oleic to palmitic acid ratio improved DM and NDF digestibility with a ratio of 60:30 palmitic to oleic compared with 80:10. In this study, the ratio of oleic to palmitic varied but the amount of palmitic was consistent between the diets. Increasing the oleic to stearic acid ratio improved DM and NDF concentration (Prom and Lock, 2021). Our study further supports the impact that FA profile has on nutrient digestibility; however, the response on NDF digestibility was different compared with prior reports, potentially the result of higher FA content of diets. There was also a parity effect on fat digestibility that indicates primiparous cows had lower FA digestibility, which has not been explored.

When conventional soybean oil diets are fed, a shift in milk FA profile occurs with a decrease in de novo (milk FA with C<16 produced in the mammary gland) and an increase in preformed (milk FA with C>16 derived from diet and adipose), ultimately resulting in reduced milk fat concentration (Boerman and Lock, 2014). The decreased lipogenesis in the mammary gland is associated with reduced gene expression of proteins and enzymes involved in FA uptake, synthesis, transport, and esterification in the mammary gland, leading to MFD (Piperova et al., 2000; Peterson et al., 2003; Harvatine and Bauman, 2006). Of note, our diets both contained a methionine analog 2-hydroxy-4-(methylthio) butanoate (**HMTBa**) product, which has been shown to reduce the shift toward alternative biohydrogenation intermediates (Baldin et al., 2018; Pitta et al., 2020). The inclusion of the HMTBa product may have reduced the potential MFD effects of HOSO supplementation in this study. As we observed increased FA digestibility with oleic acid supplementation, we would expect increased preformed milk FA to be available for incorporation into milk fat. With no effect on milk fat yield, and without measuring milk FA profile, we are not able to determine if there were milk FA profile changes consistent with modest MFD. However, our results are similar to others who have supplemented high oleic soybeans compared with conventional soybeans and observed an increase on milk fat concentration (Lopes et al., 2017; Weld and Armentano, 2018).

Oleic acid supplementation has not been shown to influence BW or BCS in mid-lactation multiparous cattle (de Souza et al., 2021b, de Souza et al., 2019; Western et al., 2020b; Prom and Lock, 2021). However, we observed an increase in BW, BCS, and fat depth with HOSO supplementation that was present in multiparous but not primiparous cattle. Primiparous cattle, having not met mature BW, have different prioritization of nutrients as they partition more energy toward body frame growth rather than body reserves (NASEM, 2021). The primiparous cows in this study showed no changes in BW or BCS when supplemented HOSO compared with multiparous cows that increased in BW and BCS. Backfat measurements via ultrasound are correlated with total body fat and can be used to estimate body composition factors (Schröder and Staufenbiel, 2006). We observed a treatment difference in both primiparous and multiparous cows with both parity groups having increased backfat depth across the longissimus dorsi when supplemented with HOSO where BCS indicated no differences in body composition in primiparous cows. The disparity depicted in the results between BCS and backfat depth between parity groups can be due to frame size and observable appearance differences (Siachos et al., 2021). A potential mechanism for increased backfat depth could be the impact of oleic acid on reducing lipolysis and increasing insulin sensitivity (Abou-Rjeileh et al., 2023). We recognize that BW and BCS measurements across a short period are challenging to interpret and do not believe that all of the differences observed in BW in multiparous cows are a result of tissue accumulation but rather variability in digestive fill at the time of weighing. We weighed at a consistent time of day, for multiple days, relative to milking and feeding for both groups to try to account for variability in digestive fill. The observed changes in BCS and fat depth support that some of the effects in BW are likely a result in allocation of energy toward body reserves.

In conclusion, supplementing soybean oil high in oleic acid did not have detrimental effects on milk production parameters, specifically milk fat synthesis. The HOSO treatment increased apparent total-tract FA digestibility, suggesting more availability of FA in the small intestine. Other apparent total-tract digestibility variables were unaffected or negatively affected by HOSO supplementation with a greater effect seen in primiparous compared with multiparous cows. Multiparous cows displayed increases in BW and BCS when fed HOSO with no changes seen in primiparous cows. However, cows in both parity groups fed HOSO demonstrated an increase in backfat depth. This combination of treatment and treatment by parity effects are an area of opportunity to further investigate how primiparous cows respond to fat supplementation.
